# Evaluating the stability of artificial sand-binding vegetation by combining statistical methods and a neural network model

**DOI:** 10.1038/s41598-023-33879-5

**Published:** 2023-04-21

**Authors:** Tonglin Fu, Xinrong Li

**Affiliations:** 1 School of Mathematics and Statistics, Longdong University, Qingyang, 745000 China; 2 Shapotou Desert Research and Experiment Station, Northwest Institute of Eco-Environment and Resources, Chinese Academy of Sciences, Lanzhou, 730000 China

**Keywords:** Ecology, Environmental sciences

## Abstract

The stability of artificial sand-binding vegetation determines the success or failure of restoration of degraded ecosystem, accurately evaluating the stability of artificial sand-binding vegetation can provide evidence for the future management and maintenance of re-vegetated regions. In this paper, a novel data-driven evaluation model was proposed by combining statistical methods and a neural network model to evaluate the stability of artificial sand-binding vegetation in the southeastern margins of the Tengger Desert, where the evaluation indexes were selected from vegetation, soil moisture, and soil. The evaluation results indicate that the stability of the artificially re-vegetated belt established in different years (1956a, 1964a, 1981a, and 1987a) tend to be stable with the increase of sand fixation years, and the artificially re-vegetated belts established in 1956a and 1964a have almost the same stability, but the stability of the artificially re-vegetated belt established in 1981a and 1987a have a significant difference. The evaluation results are reliable and accurate, which can provide evidence for the future management of artificial sand-binding vegetation.

## Introduction

The sand area in north China has the characteristics of the large area, wide distribution, and spanning several different bio-climatic zones^1^. The hazards of sandstorms and land desertification have seriously hindered ecological restoration and economic development in northern China^2^. To prevent desertification and realize the restoration of ecology, China has established artificial sand-binding vegetation on 6000,000 ha of windblown sand hazard area, which effectively improved the ecological environment, curbed the spread of land desertification, and decreased wind-sand hazards^1,3^. The practice has proven that establishing artificial sand-binding vegetation is an effective way for preventing the hazard of wind-sand and promoting regional ecological restoration. However, a series of ecological problems (e.g. new desertification appearing in previously re-vegetated desert regions, groundwater levels beginning to decline and large areas of artificial sand-binding vegetation degrading over the decades, etc.) have arisen with the implementation of a series of ecological projects^1^. Different from natural vegetation, artificial sand-binding vegetation is established with the clear purpose and function, the stability of re-vegetated ecosystems requires appropriate manual intervention, and maintenance in time^3^. Therefore, evaluating the stability of artificial sand-binding vegetation timely and accurately can provide evidence for the management and maintenance of re-vegetated ecosystems^4^.

Evaluating the stability of the ecosystem is one of the core topics in ecology, many researchers have proposed numerous methods to evaluate the stability of ecosystem^1,2,5–9^. In general, these methods can be mainly divided into dynamical system models^1,2,7^ and empirical models^5,6,8,9^. Dynamical system models, including the Lotka–Volterra model^10,11^, vegetation pattern model^12,13^, soil moisture coupled with dynamic change of vegetation^2,14^, etc., were employed to investigate the stability of the ecosystem from the perspective of dynamical system theory; The empirical methods such as fuzzy comprehensive evaluation method^5^, analytic hierarchy process^6^, Godron method^15^, variable weight evaluation method^16^, etc., comprehensively evaluated the stability of the ecosystem by using the stability indexes that closely related to the structure and function of the ecosystem. Both dynamical system models and empirical methods can be employed to evaluate the stability of ecosystems, but the theoretical results obtained by dynamical system models are difficult to be verified in reality^10–14^, and the empirical methods have the drawbacks that the weights of evaluation indexes are scored by experts^5,6,15,16^, the uncertainty of parameters may lead to inconsistency of evaluation results.

Stability is one of the basic characteristics of the ecosystem, and the definition of stability is diversified. E.g., the concepts including resistance, resilience, persistence, variability, local stability, global stability, structural stability, trajectory stability, constancy, relative stability, and set stability, etc., are used to describe ecosystem stability from different perspectives^6^. As a result, there is still no uniform standard for the measurement of ecosystem stability. This study aims to construct a data-driven hybrid evaluation model by integrating the Bootstrap technique, Monte Carlo simulation, Tagaki–Sugeno fuzzy neural network (T–S FNN), and Kruskal–Wallis (K–W) test to evaluate the stability of re-vegetated ecosystem in southeastern margins of the Tengger Desert under the assumption that the natural vegetation system is stable. The evaluation indexes are selected from vegetation, soil, and soil moisture since the vegetation, soil, and soil moisture are decisive factors for the stability of artificial sand-binding vegetation. The evaluation model is data driven and the requirement of sample size is not high, which can be applied to evaluate the stability of the re-vegetated ecosystems in other areas.

## Materials and methods

### Study site

**Figure 1:**
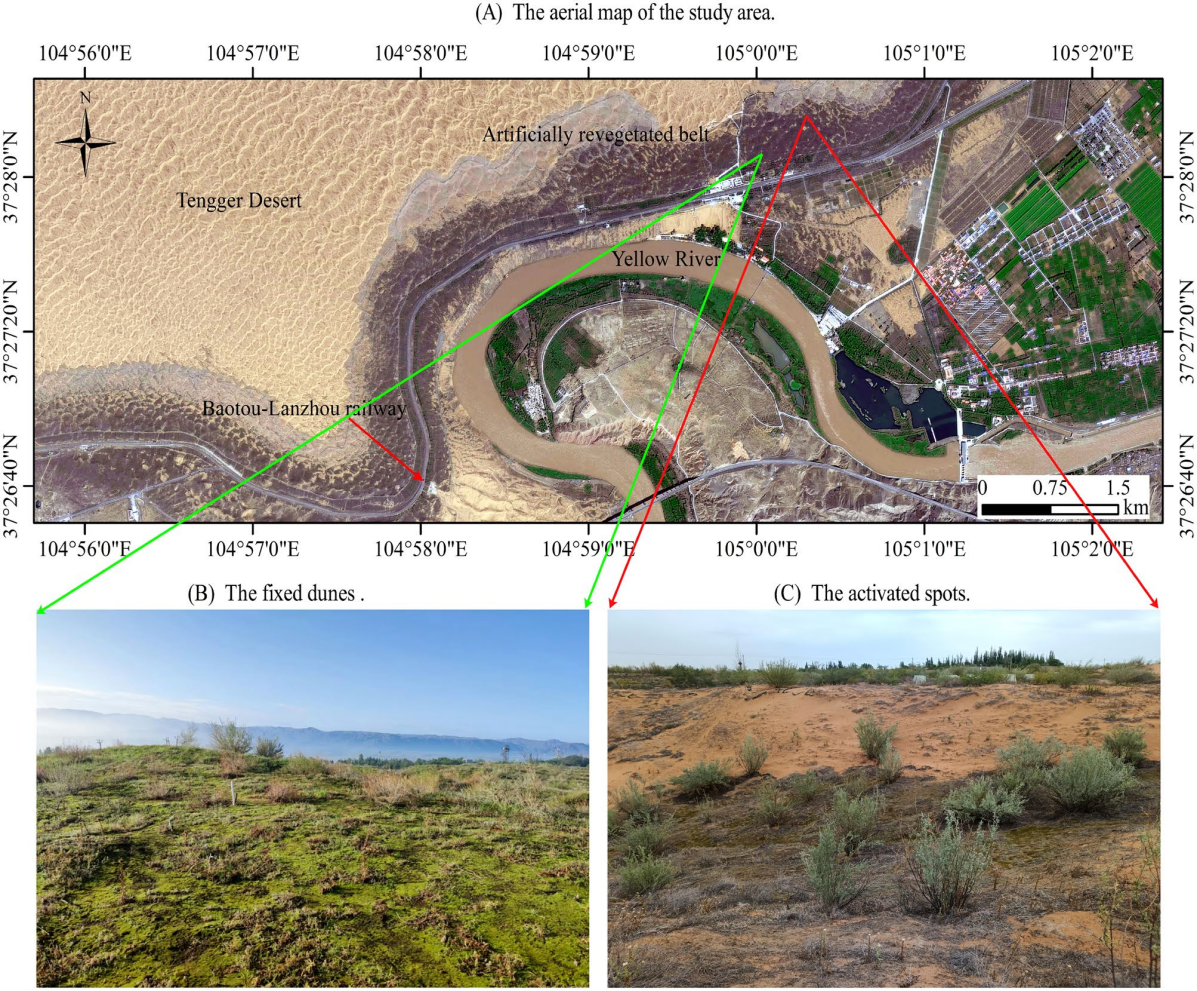
Schematic description of the study area. (**A**) The aerial map of the study area; (**B**) the fixed duns; (**C**) the activated spots in the artificially re-vegetated belt. (Using ArcGIS v. 10.8 software; Powered by ESRI “Environmental Systems Research Institute”, www.esri.com).

The study was conducted in the southeast edges of Tengger Desert (37° 32′ N, 105° 02′ E). The densely distributed trellis dunes are the main landscape type in this area (Fig. 1A). The average annual precipitation is 180.6 mm and the annual average evaporation is 2520.4 mm, the mean monthly temperatures are − 6.9 °C in January and 24.3 °C in July^1,6^. To ensure the operation of the Shapotou section of the Baotou-Lanzhou railway, the artificially re-vegetated belts were established in 1956a, 1964a, 1981a, and 1987a without irrigation, the moving dunes were fixed, plant species and the vegetation coverage have significantly increased (Fig. 1B), and a biological protective system was eventually established with a length of 16 km and a width of 200–1000 m (Fig. 1A)^1,2,6,17,18^. However, there are many "activated spots" (Fig. 1C) appeared in the artificially re-vegetated belts due to drought, wind erosion, sand burial, and other factors in the long process of succession, which seriously affect the stability and sustainability of the stability and sustainability of the re-vegetated ecosystems. To ensure the artificial sand-binding vegetation plays the best ecological and economic benefits continuously and stably, it is necessary to accurately evaluate the stability of the re-vegetated ecosystems.

### Evaluation index and data collection

Ten 10 m × 10 m quadrats were set in each artificial sand-binding vegetation belt established in 1956a, 1964a, 1981a, and 1987a as well as in the adjacent natural vegetation belt for a total of 50 quadrats, the vegetation (woody + herbaceous) coverage (%), species, crust and soil thickness (0–5 cm), soil bulk density (%), soil moisture (%), and maximum water holding capacity of surface soil (%) were observed and recorded in September 2020. The Shannon–Wiener index in the observed areas was computed by using $H = - p_{i} \ln p_{i}$. The measurement methods of other evaluation indicators are omitted since the detailed descriptions can be found in^19^. Table 1 shows the mean and standard deviation of the evaluation indexes.

**Table 1:** The mean and standard deviation of the evaluation indexes.

Evaluation index	Natural	1956a	1964a	1981a	1987a
Vegetation coverage (%)	0.48 ± 0.03	0.47 ± 0.04	0.46 ± 0.02	0.32 ± 0.06	0.26 ± 0.06
Shannon–Wiener index	2.87 ± 0.44	1.64 ± 0.37	1.52 ± 0.44	1.39 ± 0.41	0.86 ± 0.24
Soil thickness (cm)	4.87 ± 0.84	2.5 ± 0.03	2.2 ± 0.16	1.7 ± 0.02	1.4 ± 0.04
Soil bulk density (%)	1.57 ± 0.08	1.54 ± 0.02	1.47 ± 0.03	1.36 ± 0.02	1.35 ± 0.02
Soil moisture (%)	3.31 ± 0.27	2.56 ± 0.16	2.2 ± 0.38	2.1 ± 0.21	2.09 ± 0.53
Maximum water holding capacity (%)	24.16 ± 2.17	16.22 ± 1.71	16.87 ± 1.62	15.44 ± 1.35	13.38 ± 2.26

### Methods

#### Bootstrap self-sampling technique

Bootstrap self-sampling is generally used to obtain the robust estimation of the mean and standard deviation of the population by repeating random sampling from observed samples, parametric and non-parametric self-sampling are two ways to obtain Bootstrap samples, where parametric self-sampling requires the population distribution to be known in advance, but it is usually difficult to obtain the population distribution based on a limited sample. The non-parametric Bootstrap self-sampling technique can effectively overcome the defects of parametric sampling and obtain the robust estimation of parameters from small samples. In this study, the non-parametric bootstrap technique was used to estimate the mean and standard deviation of each evaluation index based on the observed data of fixed samples, that is, the mean $\mu_{i,j}$ and standard deviation $\sigma_{i,j}$ of each evaluation index in natural vegetation area and the artificial re-vegetated belt in different years (1956a, 1964a, 1981a, and 1987a) were estimated with the sample mean $\overline{X}_{ij}$ and sample standard deviation $S_{ij}$.

#### Monte Carlo simulation

Monte Carlo simulation is a well know random simulation method that can be used to generate pseudo-random numbers of a given distribution. As the measures taken by the artificial sand-binding vegetation engineering within a certain range are the same, we assumed that each evaluation index follows the uniform distribution according to the spatial self-similarity of re-vegetated ecosystems in the Monte Carlo simulation processes^1,2,19^. On the other hand, it was also reasonable to assume that each evaluation index follows normal distribution by the central limit theorem because the artificial sand-fixing vegetation may be disturbed by various random factors (e.g. micro-topography, moisture, nutrient, etc.) in the long-term succession process. Therefore, Monte Carlo simulation is a feasible and effective method to obtain sufficient samples based on the results of Bootstrap. In the Monte Carlo simulation processes, the mean and standard deviation of generated pseudo-random numbers are consistent with the estimated results of Bootstrap.

#### Tagaki–Sugeno fuzzy neural network

T–S FNN was proposed by Takagi and Sugeno in 1985 based on fuzzy set theory and fuzzy "if–then" rules^20^, it combines the advantages of a neural network model and fuzzy inference system, and has the strong adaptive ability and robustness, which is widely used in control theory, water resource assessment, and environmental management^5,8,21^. However, few scholars have applied T–S FNN to evaluate the stability of the re-vegetated ecosystems.

T–S FNN consists of four layers, including the input layer, fuzzy layer, fuzzy rule calculation layer, and output layer. The input layer is connected to the input vector $$X = [x_{1} ,x_{2} ,$$$$\cdots ,x_{k} ]^{T}$$, and each component of the input vector $$X$$ is a fuzzy variable, which is defined in the domain $$U_{i}$$ with the value $$A_{j}^{i} ,i = 1,2, \cdots ,n,j = 1,2, \cdots ,k$$. The fuzzy "if–then" rule is

$$ R^{i} :\,{\text{If}}\,x_{j} \,{\text{is}}\,A_{j}^{i} ,\,j = 1,2, \ldots ,k,\,{\text{then}}\,y = p_{0}^{i} + p_{1}^{i} x_{1} + p_{2}^{i} x_{2} + \cdots + p_{k}^{i} x_{k} $$

The membership function of the input value $x_{i}$ is determined by

1 $$ \mu (A_{j}^{i} ) = \exp \left\{ { - (x_{j} - c_{j}^{i} )^{2} /b_{j}^{i} } \right\},\,\;i = 1,2, \ldots ,n,\;j = 1,2, \ldots ,k $$

where $c_{j}^{i}$ and $b_{j}^{i}$ denote the center and width of the membership function, respectively. The applicability $w_{i}$ of each fuzzy rule is calculated by using the continuous multiplication operator, that is

2 $$ w_{i} = \mu_{{A_{j}^{1} }} (x_{1} )\mu_{{A_{j}^{2} }} (x_{{2}} ) \ldots \mu_{{A_{j}^{k} }} (x_{k} ),\;i = 1,2, \cdots ,n. $$

The output of T–S FNN is

3 $$ y_{i} = \sum\limits_{i = 1}^{n} {w^{i} (p_{0}^{i} + p_{1}^{i} x_{1} + p_{2}^{i} x_{2} + \cdots + p_{k}^{i} x_{k} )/} \sum\limits_{i = 1}^{n} {w^{i} } ,i = 1,2, \cdots ,n. $$

which can be regarded as evaluation results of the stability of artificial sand-binding vegetation, the smaller the $y_{i}$ value, the more stable the system is.

**Figure 2:**
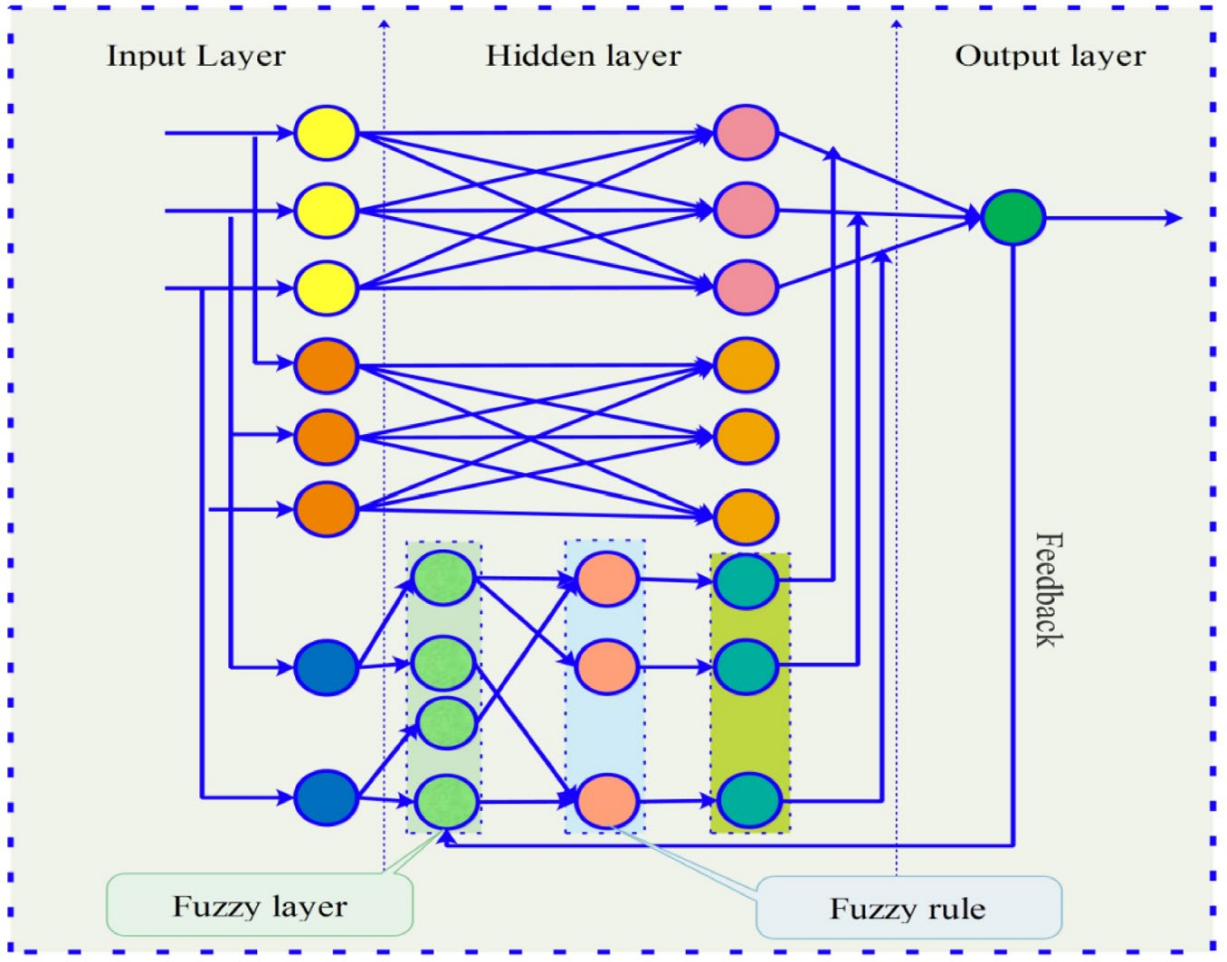
The diagram of T–S FNN.

The error function is

4 $$ E = \frac{1}{2}\sum\limits_{i = 1}^{r} {(y_{di} - y_{i} )^{2} } , $$

where $y_{di}$ and $y_{i}$ denote the expected output and the actual output, respectively. The adjustment parameters, including $p_{j}^{i}$, $c_{j}^{i}$, and $b_{j}^{i}$, are computed by using the error backpropagation algorithm and the first order gradient optimization algorithm,

5 $$ p_{j}^{i} (k + 1) = p_{j}^{i} (k) - \beta \frac{\partial E}{{\partial p_{j}^{i} }}, $$

6 $$ c_{j}^{i} (k + 1) = c_{j}^{i} (k) - \beta \frac{\partial E}{{\partial c_{j}^{i} }}, $$

7 $$ b_{j}^{i} (k + 1) = b_{j}^{i} (k) - \beta \frac{\partial E}{{\partial b_{j}^{i} }}, $$

where

8 $$ \frac{\partial E}{{\partial p_{j}^{i} }} = (y_{di} - y_{i} )w^{i} x_{j} /\sum\limits_{i = 1}^{n} {w^{i} } ,\;i = 1,2, \cdots ,n,\;j = 1,2, \cdots ,k, $$

and $\beta > 0$ is the learning rate of T–S FNN. Figure 2 shows the diagram of T–S FNN.

To overcome the drawbacks of insufficient training and over-training in the neural network model, the Nash–Sutcliffe coefficient of efficiency (NSCE), mean absolute percentage error (MAPE), root mean squared error (RMSE), and mean absolute error (MAE), were used to determine the optimal training times of T–S FNN. Table 2 shows the definition of NSCE, MAPE, RMSE, and MAE, where NSCE was a positive indicator, and MAPE, RMSE, and MAE were negative indicators. The closer the value of NSCE is to 1, and the smaller the value of MAPE, RMSE, and MAE is, the better the training effect of the model will be. The trained T–S FNN is used to evaluate the stability of the artificial sand-binding vegetation.

**Table 2:** The definition of NSCE, MAPE, RMSE, and MAE.

NSCE	Nash–Sutcliffe coefficient of efficiency	$NSCE = 1 - \frac{{\sum\nolimits_{i = 1}^{n} {\left( {x_{i} - \widehat{x}_{i} } \right)^{2} } }}{{\sum\nolimits_{i = 1}^{n} {\left( {x_{i} - \overline{x} } \right)^{2} } }}$
MAPE	Mean absolute percentage error	$MAPE = \frac{{1}}{n}\sum\nolimits_{i = 1}^{n} {\left| {\frac{{x_{i} - \widehat{x}_{i} }}{{x_{i} }}} \right|} \times 100\%$
RMSE	Root mean squared error	$RMSE = \sqrt {\frac{{1}}{n}\sum\nolimits_{i = 1}^{n} {\left( {x_{i} - \widehat{x}_{i} } \right)^{2} } }$
MAE	Mean absolute error	$MAE = \frac{{1}}{n}\sum\nolimits_{i = 1}^{n} {\left| {x_{i} - \widehat{x}_{i} } \right|}$

#### Kruskal–Wallis test

The K–W test is a non-parametric test with the advantage of without the need to meet the normality assumption^22^. The null hypothesis of the K–W test is that the evaluation results have no significant difference with the significance level $\alpha = 0.05$. If the P value of the K–W test is less than the significance level $\alpha$, the null hypothesis is rejected; Otherwise, accept the null hypothesis. In this study, the K–W test was used to determine the significant difference in the evaluation results.

#### Hybrid evaluation model

In this study, the Bootstrap technique, Monte Carlo simulation, T–S FNN, and K–W test were integrated to construct the hybrid evaluating model to evaluate the stability of re-vegetated ecosystem based on the observed data of fixed quadrats in the artificially re-vegetated belt established in different years (1956a,1964a,1981a, and 1987a) and natural vegetation area in the southeastern margin of the Tengger Desert. As the vegetation, soil, and soil moisture determine the stability of non-irrigated artificial sand-binding vegetation when the annual average rainfall is unchanged^1,2^, the vegetation coverage (%), Shannon–Wiener index, crust and soil thickness (cm), soil bulk density (%), soil moisture (%), and maximum water holding capacity of surface soil (%) were selected as the evaluation indexes to represent vegetation, soil and soil moisture in the evaluation process. In addition, We assumed that the natural vegetation system is stable because the natural vegetation system has adapted to the regional climatic and soil conditions in the long course of vegetation succession. The corresponding observation indexes of natural vegetation were regarded as the benchmarks to determine the membership degree of the evaluation index. MATLAB software (R2019a, MathWorks, USA) is utilized to implement all the computing processes. The main steps of the data-driven evaluating model are as follows:

- Step 1. Selecting the evaluation indexes from the aspects including the vegetation, soil, and soil moisture since these factors determine the stability of artificial sand-binding vegetation.
- Step 2. The Bootstrap technique was used to obtain robust estimates of the mean and variance of the population of evaluation indexes based on the observed data.
- Step 3. Monte Carlo simulation was employed to generate the training and testing set of T–S FNN under the assumption that each evaluation index obeys uniform distribution or normal distribution, respectively.
- Step 4. The indexes of undisturbed natural vegetation in the same area were taken as the reference to determine the membership degree of the evaluation index, and the T–S FNN was used to evaluate the stability of the artificial sand-binding vegetation.
- Step 5. The K–W test is used to determine whether there is significant differences in the evaluation results of T–S FNN.

## Results

**Figure 3:**
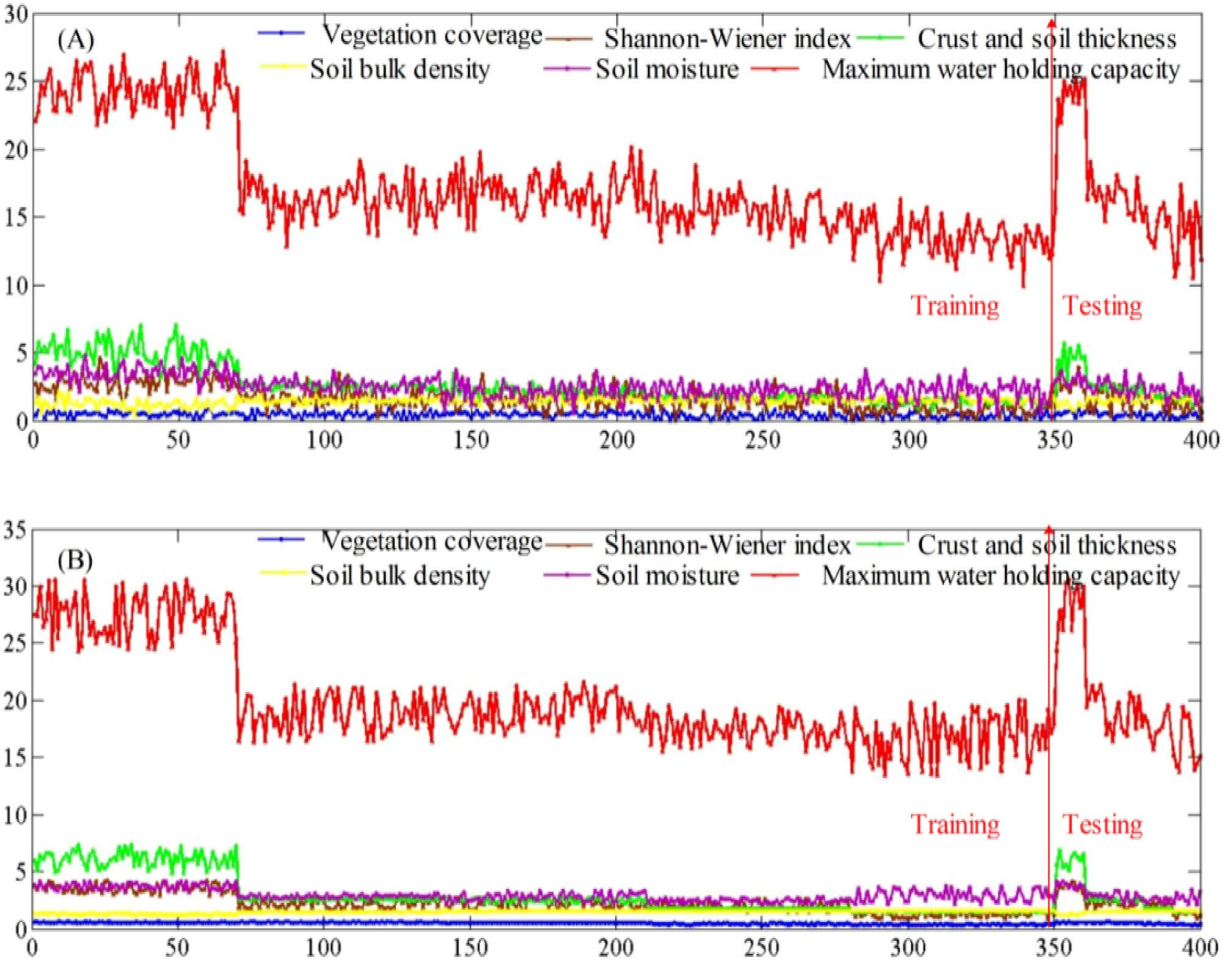
The Monte Carlo simulation results of evaluation indexes. (**A**) The evaluation indexes are uniformly distributed; (**B**) The evaluation indexes are normally distributed.

Table 3 shows the Bootstrap estimation of the population mean and standard deviation of the evaluation indexes, where the sampling number is 1000. As the distribution of each evaluation index is unknown, we assumed that each evaluation index obeys uniform distribution and normal distribution, respectively. Monte Carlo simulation was used to randomly generate 400 samples (Fig. 3) that obey uniform distribution and normal distribution, respectively (Supplementary information file). The mean and standard deviation of generated pseudo-random numbers were determined according to Table 3. The first 350 samples were used to train T–S FNN, and the last 50 samples were used for testing. The ratio between the training and testing sets was 7:1. The input node of T–S FNN was 6, the number of hidden layer nodes was 12, and the output node was 1.

**Table 3:** The Bootstrap estimation of the population mean and standard deviation of each evaluation index.

Evaluation index	Natural	1956a	1964a	1981a	1987a
Vegetation coverage (%)	0.54 ± 0.05	0.52 ± 0.05	0.47 ± 0.04	0.31 ± 0.06	0.24 ± 0.05
Shannon–Wiener index	2.75 ± 0.20	1.60 ± 0.27	1.54 ± 0.21	1.35 ± 0.16	0.96 ± 0.13
Soil thickness (cm)	4.54 ± 0.26	2.52 ± 0.04	2.28 ± 0.08	1.72 ± 0.03	1.53 ± 0.05
Soil bulk density (%)	1.56 ± 0.07	1.56 ± 0.05	1.40 ± 0.06	1.37 ± 0.05	1.35 ± 0.03
Soil moisture (%)	2.99 ± 0.11	2.59 ± 0.12	2.37 ± 0.20	2.01 ± 0.13	2.05 ± 0.18
Maximum water holding capacity (%)	24.05 ± 0.30	16.59 ± 0.32	16.23 ± 0.37	14.34 ± 0.37	13.57 ± 2.26

To determine the optimal training times and prevent insufficient training or over-training of T–S FNN, the training times of T–S FNN were set as 500, 1000, 1500, and 2000, respectively. The NSCE, MAPE, RMSE, and MAE were used to evaluate the training effect. The results of NSCE, MAPE, RMSE, and MAE at different training times under the assumption that each evaluation index obeys uniform or normal distribution are shown in Table 4. The trained T–S FNN was employed to evaluate the stability of artificially re-vegetated belts in different years (1956a, 1964a, 1981a, and 1987a). The evaluation results of different training times are shown in Tables 5 and 6, respectively.

**Table 4:** The NSCE, MAPE, RMSE and MAE of T–S FNN with different training times.

Distribution function	Training times	500	1000	1500	2000
Uniformly distributed	NSCE	0.9070	0.9546	**0.9913**	0.9655
	MAPE	0.0289	0.0218	**0.0013**	0.0159
	RMSE	0.2234	0.1604	**0.0734**	0.1415
	MAE	0.1636	0.1234	**0.0551**	0.1051
Normally distributed	NSCE	0.7583	0.8100	**0.9298**	0.8363
	MAPE	0.0490	0.0067	**0.0005**	0.0055
	RMSE	0.3318	0.3131	**0.2019**	0.2943

Significant values are in [bold].

**Table 5:** The evaluation results of T–S FNN under different training times under the assumption that each evaluation index is uniformly distributed.

Training times	Evaluation object	Evaluation results										Mean	Std
500	1956a	0.6843	0.9035	1.1188	0.8087	1.1471	1.0973	0.9818	1.2268	1.2627	0.9667	1.0198	0.1852
	1964a	0.9736	1.2394	1.2187	1.2372	1.3791	1.4029	1.0458	1.2190	1.3858	1.3951	1.2496	0.1490
	1981a	2.4861	1.9154	1.4800	1.8688	2.0820	1.6333	2.2600	2.6214	1.8135	1.8188	1.9979	0.3642
	1987a	2.7595	2.5014	2.6722	2.6680	2.3956	2.9335	2.7891	2.3408	3.1381	3.2054	2.7404	0.2906
1000	1956a	0.9576	1.0280	1.0784	1.0958	1.0547	0.9954	0.9618	1.1009	1.0944	0.8610	1.0228	0.0786
	1964a	0.9525	0.9596	1.1227	1.2665	1.2358	1.3351	0.9501	1.0228	1.3574	1.3073	1.1510	0.1684
	1981a	2.2558	2.0819	1.7062	1.8615	2.1333	1.8177	2.2559	2.5329	1.9940	1.9332	2.0572	0.2472
	1987a	2.8809	2.6298	2.8174	2.7561	2.5583	3.0163	2.8044	2.6152	3.0284	3.1186	2.8225	0.1902
1500	1956a	1.0439	1.0168	1.0386	1.0444	1.0663	0.9854	0.9876	1.0189	1.0474	0.9656	**1.0215**	0.0327
	1964a	1.0133	0.9554	1.1146	1.2198	1.0601	1.2253	0.9632	0.9596	1.2158	1.2103	**1.0937**	0.1173
	1981a	2.3590	2.2876	2.2745	2.2389	2.2980	2.2795	2.3773	2.4074	2.3267	2.2892	**2.3138**	0.0526
	1987a	3.0835	2.9117	3.0048	2.9986	2.9273	3.1612	2.9559	3.0445	2.9080	3.0813	**3.0077**	0.0846
2000	1956a	1.0387	0.9533	0.9815	1.0664	1.0392	0.8870	0.8961	0.9840	1.0236	0.9644	0.9834	0.0604
	1964a	0.9070	0.8552	1.1059	1.2391	1.0527	1.2442	0.8682	0.8329	1.2931	1.2374	1.0636	0.1849
	1981a	2.1576	2.1962	1.8133	1.9567	2.1354	1.9607	2.2307	2.4747	2.0766	2.0324	2.1034	0.1821
	1987a	2.9298	2.7630	2.8348	2.8450	2.6483	3.0234	2.8364	2.8062	2.9606	3.0661	2.8714	0.1254

Significant values are in [bold].

**Table 6:** The evaluation results of T–S FNN under different training times under the assumption that each evaluation index is normally distributed.

Training times	Evaluation object	Evaluation results										Mean	Std
500	1956a	1.1440	1.1234	1.1190	1.1384	0.9605	1.0203	1.0775	1.0532	1.1051	0.9890	1.0730	0.0649
	1964a	1.0064	1.0287	1.0647	1.1739	0.9923	1.1556	0.9874	0.9521	1.1748	1.0826	1.0618	0.0824
	1981a	1.6325	1.2672	1.4069	1.3576	1.4374	1.4191	1.5915	1.8697	1.4634	1.4059	1.4851	0.1713
	1987a	2.4709	1.4657	2.3386	1.7805	1.8227	2.3728	1.5687	1.7184	2.2732	2.0427	1.9854	0.3621
1000	1956a	1.1324	1.0928	1.1371	1.1326	1.1725	1.1361	1.1147	1.1494	1.1356	1.0849	1.1288	0.0257
	1964a	1.1632	1.0652	1.2766	1.5048	1.2070	1.4885	1.1268	1.1226	1.5219	1.3412	1.2818	0.1731
	1981a	2.4444	2.2871	2.2885	2.2220	2.3644	2.3475	2.4645	2.5868	2.3978	2.3214	2.3724	0.1057
	1987a	2.9266	2.7048	2.8853	2.7942	2.7547	2.9237	2.7353	2.8258	2.8533	2.8565	2.8260	0.0774
1500	1956a	1.2099	1.2126	1.2368	1.2223	1.2558	1.1984	1.1776	1.2064	1.2655	1.2198	**1.2205**	0.0263
	1964a	1.2043	1.1374	1.3243	1.6343	1.3155	1.5905	1.2046	1.1981	1.6762	1.4500	**1.3735**	0.2005
	1981a	2.3679	2.4933	2.1360	2.0908	2.4635	2.3716	2.5142	2.6427	2.4875	2.3051	**2.3873**	0.1722
	1987a	2.9547	2.7186	2.9269	2.8186	2.8002	2.9497	2.7102	2.8086	2.8341	2.8837	**2.8405**	0.0877
2000	1956a	1.3776	1.2975	1.2779	1.3484	1.0553	1.0945	1.2513	1.1421	1.2608	1.2661	1.2371	0.1062
	1964a	1.0012	1.2201	1.0594	1.2523	1.0879	1.1765	1.0488	0.9717	1.2608	1.2437	1.1322	0.1107
	1981a	1.8582	1.3369	1.4481	1.4414	1.5642	1.4968	1.8752	2.1776	1.6009	1.5672	1.6366	0.2567
	1987a	2.7275	1.7655	2.6302	2.1297	2.1884	2.6977	1.9875	2.2202	2.5364	2.4866	2.3370	0.3263

Significant values are in [bold].

As shown in Table 4, when the training times of TS-FNN are 1500, NSCE reaches the maximum, and MAPE, RMSE, and MAE close to the maximum, indicating that the optimal training times of T–S FNN is 1500. Tables 5 and 6 show that the evaluation results of artificial sand-binding vegetation in different years became accurate with the increase in training times, and the standard deviation of the evaluation results is the smallest if the training number is 1500. The mean of the stability evaluation results of artificial sand-binding vegetation in different years (1956a,1964a,1981a, and 1987a) are 1.0215, 1.0937, 2.3138, and 3.0077 under the assumption that each evaluation index is uniformly distributed (Table 5); The mean of evaluation results of the artificial sand-binding vegetation with the same number of training times are 1.2205, 1.3735, 2.3873, and 2.8405 under the assumption that each evaluation index is normally distributed (Table 6). Therefore, we can conclude that the stability of artificial sand-binding vegetation in different years is: artificial sand-binding vegetation established in 1956a > artificial sand-binding vegetation established in 1964a > artificial sand-binding vegetation established in 1981a > artificial sand-binding vegetation established in 1987a, suggesting that the stability of the artificially re-vegetated belt established in different years (1956a, 1964a, 1981a, and 1987a) tend to be stable with the increase of sand fixation years.

K–W test was employed to determine whether there is significant differences in the evaluation results of artificial sand-binding vegetation established in different years (1956a, 1964a, 1981a, and 1987a) (Tables 5, 6) with the significance level $\alpha$ of 0.05. The P value of the evaluation results of artificial sand-binding vegetation belts established in 1956a and 1964a are 0.2568 and 0.3643 under the assumption that the population distribution of evaluation indexes are uniformly distributed or normally distributed (Table 7), which are all greater than the significance level $\alpha$, indicating that there is no difference in the stability evaluation results of the artificial sand-binding vegetation belts between 1956 and 1964a, that is, the artificial sand-binding vegetation belts of 1956a and 1964a have almost the same stability; The P value of the evaluation results of artificial sand-binding vegetation belts established in 1981a and 1987a are all close to 0.0002, which significantly less than the significance level $\alpha$, indicating that the stability of artificial sand-binding vegetation belts established in 1981a and 1987a have significant difference (Table 7).

**Table 7:** The results of the K–W test.

Training times	Normally distributed	P Value	Difference	Uniformly distributed	P Value	Difference
500	1956a vs 1964a	0.0126	Yes	1956a vs 1964a	0.8206	No
	1982a vs 1987a	0.0006	Yes	1982a vs 1987a	0.0019	yes
1000	1956a vs 1964a	0.1736	No	1956a vs 1964a	0.0588	No
	1982a vs 1987a	0.0004	Yes	1982a vs 1987a	0.0001	Yes
1500	1956a vs 1964a	**0.3643**	**No**	1956a vs 1964a	**0.2568**	**No**
	1982a vs 1987a	**0.0014**	**Yes**	1982a vs 1987a	**0.0001**	**yes**
2000	1956a vs 1964a	0.4057	No	1956a vs 1964a	0.0233	Yes
	1982a vs 1987a	0.0001	Yes	1982a vs 1987a	0.0006	Yes

Significant values are in [bold].

## Discussion

### Model testing

- The traditional evaluation methods can be mainly divided into dynamical system models and empirical models. However, the theoretical results obtained by dynamical system models are difficult to be verified in reality, and the empirical methods have the drawbacks that the weights of evaluation indexes are scored by experts, and the uncertainty of parameters may lead to inconsistency of evaluation results. In addition, evaluating the stability of an ecosystem comprehensively and systematically requires a large number of observed variables and data, which will lead to huge costs. Therefore, how to evaluate the stability of re-vegetated ecosystems with limited observational data is a challenging problem.

In this study, the bootstrap technique was employed to obtain the robust estimation of the mean and standard deviation of each evaluation index based on the observed data, which provides a standard for Monte Carlo simulation. As mentioned above, Monte Carlo simulation was employed to generate enough pseudo-random numbers for training the T–S FNN under the different assumptions, which makes it possible to evaluate the stability of artificial sand-binding vegetation systems by using the machine learning model. As T–S FNN combines the advantages of the neural network model and fuzzy inference system, and the evaluating results clearly show the stability score in each quadrat in each artificial sand-binding vegetation belt established in different years, which provides a basis for the precision management of re-vegetated ecosystems in the study area. Finally, the K–W test was employed to determine the difference in stability of artificial sand-binding vegetation established in different years (1956a, 1964a, 1981a, and 1987a), and the result show that the artificial sand-binding vegetation belts established in 1956a and 1964a have almost the same stability, but the stability of artificial sand-binding vegetation belts established in 1981a and 1987a have a significant difference. This conclusion is consistent with the views of other scholars^1,2,6,7^.

Although the application of the proposed evaluation model requires a large number of pseudo-random data, and the evaluation results are completely determined by the input variables and the training times of T–S FNN, the proposed evaluation model has its advantages. E.g., compared with the traditional evaluation methods, the proposed evaluation model is data-driven, which effectively overcomes the drawbacks that the theoretical results of dynamical system models are difficult to be verified in reality, and the weight of evaluation index in empirical methods exists in the uncertainty. The proposed evaluation model has good universality, which can be employed to evaluate the stability of artificial sand-binding vegetation with limited observational data in other bioclimatic zones.

### The stability of the revegetated ecosystems

The stability of the artificial sand-binding vegetation is a necessary condition for the sustainability of the re-vegetated ecosystems, which determines the rise and fall of the re-vegetated ecosystem, and relates to the prospective function and skopos. Before the establishment of the artificial sand-binding vegetation system, the native shrub coverage in the southeastern margin of the Tengger Desert was below 1%. With the establishment of the artificial sand-binding vegetation, the shrub coverage reached 33% at most after 15a, and the coverage of the herbs did not exceed 5%. With the increase of sand fixation years and the continuous colonization of herbaceous species, shrub coverage gradually decreased to 6%-10%, while herbaceous species increased by over 30%^1,2,6,17,18^. The main artificial sand-binding shrubs (e.g. *Caragana korshinskii, Caragana microphylla, Calligonum mongolicum, Hedysarum scoparium, Atraphaxis Bracteata and Artemisia ordosica,* etc.) have been gradually replaced by natural herbs (e.g. *Bassia dasyphylla, A. capillaries, Aristida Adscensionis, Eragrostis minor, Salsola Ruthenica, Setaria Viridis, Stipa Glareosa, Artemisia blepharolepis, Corpermum declinatum steph. ex Iljin, Agriophyllum, Echinops gmelinii, Scorzonera divargos, Allium mongolicum, Euohorbia humifusa Willd* etc.)^18^. This succession can be regarded as the result of competition between herbs and shrubs for limited water resources.

Soil moisture is the driving force and key a-biotic limiting factor for the succession of artificial sand fixation vegetation. Soil water controls the ecological process of artificial sand-binding vegetation in arid sand areas^1–3^. The establishment of artificial sand-binding vegetation changed the original eco-hydrological process of mobile dunes and promoted the water-holding capacity of surface soil^1,7,17,18^. With the increase of sand fixation years, the dynamic change of soil moisture changed the distribution pattern of vegetation. The coverage of sand fixation shrubs and deep soil moisture reached a new equilibrium state^1,2^. According to the niche differentiation theory^2,6,17^, deep soil moisture restricts shrub coverage, while shallow soil moisture affects herb coverage.

The restoration of soil is the most fundamental indicator to measure the success of ecological reconstruction and restoration in arid sand areas. After the mobile dune was fixed with grass squares, the biological soil crust was successively formed on the sand surface with *cyanobacteria*, *lichens,* and *mosse*s as the dominant crust, the formation of topsoil was effectively promoted by the extensive colonization of biological soil crust^7,17^. With the increase of sand fixation years, the content of organic matter in soil increased. The improvement of surface soil provides a suitable habitat for the settlement and reproduction of soil microorganisms and soil micro-fauna^1,18^. Due to the continuous accumulation of atmospheric falling dust and humus on the surface soil, the formation of the surface soil in sand areas is promoted, the aggregate structure of the surface soil is increased, the bulk density of soil is reduced, and the water retention ability of soil is improved^1,2,6,7^. The effective water that can be utilized by the shallow root herb is increased, and a complex community with multiple layers including shrubs, herbs, mosses, lichens and algae is gradually formed in the artificial sand-binding vegetation area^6,17,19^.

## Conclusions

Constructing data-driven hybrid models can overcome the defects of mathematical models and empirical models effectively. In this paper, a data-driven evaluation model based on the Bootstrap technique, Monte Carlo simulation, T–S FNN, and K–W test was proposed to evaluate the stability of re-vegetated ecosystems in the Shapotou section of Baotou-Lanzhou railway under the assumption that the undisturbed natural vegetation is stable. The evaluation results show that the stability of the artificial sand-binding vegetation belts established in different years (1956a, 1964a, 1981a, and 1987a) tends to be stable with the increase of sand fixation years, and the artificially re-vegetated belt of 1956a and 1964a have almost the same stability, but the stability of the artificially re-vegetated belt between 1981 and 1987a have a significant difference. The evaluation model is data-driven, and the evaluation results depend on the inherent structure of the model. Therefore, the research method in this paper is also applicable to evaluate the stability of other ecosystems.

## Supplementary Information

Supplementary Information.

## Data Availability

All data analyzed or generated during this study are included in Supplementary information, and are available from the corresponding authors upon reasonable request.
